# UMF-078: A modified flubendazole with potent macrofilaricidal activity against *Onchocerca ochengi *in African cattle

**DOI:** 10.1186/1756-3305-1-18

**Published:** 2008-06-20

**Authors:** Barend M deC Bronsvoort, Benjamin L Makepeace, Alfons Renz, Vincent N Tanya, Lawrence Fleckenstein, David Ekale, Alexander J Trees

**Affiliations:** 1 Veterinary Parasitology, Liverpool School of Tropical Medicine/Faculty of Veterinary Science, University of Liverpool, Pembroke Place, Liverpool, UK; 2 Fachgebiet Parasitologie, Universität Hohenhiem, Germany; 3 Institut de Recherche Agricole pour le Développement, Wakwa, Cameroon; 4 College of Pharmacy, University of Iowa, Iowa City, IA 52242, USA; 5 The Roslin Institute and Royal (Dick) School of Veterinary Studies, University of Edinburgh, Easter Bush Veterinary Centre, Roslin, Midlothian, EH25 9RG, UK; 6 Universität Tübingen, Friedhofstrasse 73, 72074 Tübingen, Germany; 7 Technical Adviser No.1, Ministry of Scientific Research and Innovation, P.O. Box 1457, Yaoundé, Cameroon

## Abstract

**Background:**

Human onchocerciasis or river blindness, caused by the filarial nematode *Onchocerca volvulus*, is currently controlled using the microfilaricidal drug, ivermectin. However, ivermectin does not kill adult *O. volvulus*, and in areas with less than 65% ivermectin coverage of the population, there is no effect on transmission. Therefore, there is still a need for a macrofilaricidal drug. Using the bovine filarial nematode *O. ochengi *(found naturally in African cattle), the macrofilaricidal efficacy of the modified flubendazole, UMF-078, was investigated.

**Methods:**

Groups of 3 cows were treated with one of the following regimens: (a) a single dose of UMF-078 at 150 mg/kg intramuscularly (im), (b) 50 mg/kg im, (c) 150 mg/kg intraabomasally (ia), (d) 50 mg/kg ia, or (e) not treated (controls).

**Results:**

After treatment at 150 mg/kg im, nodule diameter, worm motility and worm viability (as measured by metabolic reduction of tetrazolium to formazan) declined significantly compared with pre-treatment values and concurrent controls. There was abrogation of embryogenesis and death of all adult worms by 24 weeks post-treatment (pt). Animals treated at 50 mg/kg im showed a decline in nodule diameter together with abrogated reproduction, reduced motility, and lower metabolic activity in isolated worms, culminating in approximately 50% worm mortality by 52 weeks pt. Worms removed from animals treated ia were not killed, but exhibited a temporary embryotoxic effect which had waned by 12 weeks pt in the 50 mg/kg ia group and by 24 weeks pt in the 150 mg/kg ia group. These differences could be explained by the different absorption rates and elimination half-lives for each dose and route of administration.

**Conclusion:**

Although we did not observe any signs of mammalian toxicity in this trial with a single dose, other studies have raised concerns regarding neuro- and genotoxicity. Consequently, further evaluation of this compound has been suspended. Nonetheless, these results validate the molecular target of the benzimidazoles as a promising lead for rational design of macrofilaricidal drugs.

## Background

Human onchocerciasis or river blindness, caused by the filarial nematode *Onchocerca volvulus*, is endemic in 34 countries in Africa, the Americas and the Yemen, infecting approximately 37 million people with a further 90 million people considered to be at risk of infection in Africa [1]. Beginning in 1974, control of the vector (*Simulium damnosum sensu lato*) was the principle approach used by the highly successful Onchocerciasis Control Programme (OCP) in 11 endemic countries of West Africa, although ivermectin distribution played a key role from 1987 [2]. The OCP was phased-out in 2002 and has been superseded by the African Programme for Onchocerciasis Control (APOC), which applies community-directed treatment with ivermectin as an almost exclusive control method in a further 19 endemic African countries. Ivermectin is currently supplied free-of-charge by Merck & Co. under their Mectizan^® ^Donation Programme [3,4].

Ivermectin is an extremely effective microfilaricide, rapidly removing microfilariae (mf) from the skin of infected patients and thus preventing the development of blindness and relieving skin irritation [5-7]. However, despite its cumulative sterilising effect on the female worm, ivermectin does not appear to exhibit significant macrofilaricidal activity [8-11]. Furthermore, in order to prevent sustained transmission, annual treatment coverage must exceed 65% of the population [12]. Following analyses of the post-control situation in the OCP region, there is a consensus that onchocerciasis cannot be eradicated in Africa using currently available methods alone [13,14].

The development of a safe, effective macrofilaricide suitable for mass treatment, preferably as a single oral dose, was the goal of the World Health Organisation's (WHO) MACROFIL programme, which also received support from OCP and APOC. Such a drug would be able to break the transmission cycle and thus reduce the number of years of treatment necessary to permanently eliminate onchocerciasis as a public health problem in Africa.

There is little published information on the anthelminthic properties of the modified flubendazole, UMF-078, but it is has been shown to have both micro- and macrofilaricidal activity against *Acanthocheilonema viteae *and *Litomosoides sigmodontis *in jirds [15]. However, flubendazole itself has been studied quite extensively. It has been shown to be both micro- and macrofilaricidal depending on the formulation and the route of administration [16-18]. Using *Onchocerca ochengi*, a natural parasite of cattle that is endemic in the Adamawa province of Cameroon [19], the efficacy of UMF-078 was assessed *in vivo *by the intramuscular or intraabomasal routes at two dosage rates. This species is the closest existent relative of *O. volvulus *[20,21] and forms palpable, intradermal nodules which can be removed under local anaesthetic. Consequently, it was adopted as a tertiary drug screen for macrofilaricides by WHO [22-24].

## Materials and methods

### Animals

Fifteen 4–6 year old Gudali cows (*Bos indicus*), each with more than 20 palpable *O. ochengi *nodules in the ventral abdominal skin, were purchased from local markets in the Adamawa Province of Cameroon. They were kept on pasture at the Institut de Recherche Agricole pour le Développement (IRAD), formerly the Institut de Recherche Zootechniques et Vétérinaires (IRZV), Wakwa, 10 km SW of Ngaoundéré. This location is at an altitude of 1000 m where the annual transmission potential (ATP) for *O. ochengi *is low [approximately 600 infective larvae per animal per year (Renz and Bronsvoort, unpublished observations)].

Twenty *O. ochengi *nodules per animal were identified, their locations noted, and their periphery marked by tattoo ink. Subsequently, a microchip transponder (Identichip^®^, Animalcare Ltd., York, UK) was subcutaneously implanted adjacent to each nodule. Each transponder carried a unique 10-digit number that was visualised using a hand-held reader. In previous experiments, these microchips remained in position for at least 2 years and enabled individual nodules to be identified consistently.

### Chemotherapy

UMF-078, methyl(+/-)5-[α-amino-4 fluorobenzyl]benzimidazole-2-carbamate, was provided as a white desiccated powder. This was suspended in peanut oil (Sigma UK) at a final concentration of 180 mg/ml.

The cattle had a mean weight of 316 kg (SD = 32 kg) and were ranked according to nodule load per animal (mean = 63 nodules; SD = 39 nodules). The four treatment and one control groups were randomly numbered 1–5 and the highest ranking animal randomly assigned to a group. The remaining cattle (in rank order) were then assigned in turn to each treatment group (in order of their number) to give a similar mean number and range of nodules in each group of three animals.

Intramuscular (im) injections (150 mg/kg or 50 mg/kg) were delivered in the gluteal and/or triceps muscles with a maximum of 50 ml of the suspension per site. Intraabomasal (ia) injections (150 mg/kg or 50 mg/kg) were administered into the lumen of the pyloric region of the abomasum following a right-sided laparotomy and exteriorisation of the pylorus [25]. The wounds were closed using monofilament nylon mattress sutures and the animals were treated for a week with Duplocillin LA^® ^(Intervet, UK). All animals recovered without incident.

### Observations

Microfilarial densities were determined as previously described (Renz et al., 1995; Tchakouté et al., 1999). In brief, superficial skin biopsies were obtained from the ventral mid-line (on the same morning for all groups) and incubated for 24 h in 0.5 ml antibiotic-supplemented Roswell Park Memorial Institute medium (RPMI 1640, Sigma UK). The biopsies were then digested in 0.5% collagenase for 24 hours. Viable mf that had emerged into the medium, together with residual mf present in digested skin, were counted by microscopy and total densities expressed per 100 mg of skin. The geometric mean number of mf per 100 mg of skin from the three biopsies for each animal was calculated using the log+1 transformation. The mean per group was calculated in a similar way.

Four nodules were removed per animal under local anaesthetic at each time-point. If a nodule had apparently resolved, its position was localised by microchip and the incision was guided by the peripheral tattoo.

All each time-point, nodules were examined by a technician blinded to the treatment group by the triple assay described by Renz *et al*. [24] with some modification. After trimming the nodule, the capsule was incised and the adult worm squeezed out into 200 μl PBS on a depression slide and males picked out. The first 5 mm of the anterior end of the females was incised. Male and female worms were incubated in 200 μl of RPMI in microtitre plate. After incubation (30 minutes, 37°C), the motility of the male worms and female anterior ends (mean length ± SD = 8.3 ± 2.6 mm) was scored on a 3 point scale (0 = no movement after 30 seconds of observation; 1 = weak, slow or intermittent movement; 2 = vigorous movement).

Individual worm viability was assessed by the metabolic reduction of 3-(4,5-dimethylthiazol-2-yl)-2,5-di-phenyltetrazolium bromide (MTT) to formazan [24,26]. Worms were incubated for 1 hour in MTT and then the colour was leached out of the worms by 1/2 hour incubation in DMSO. The optical density of the formazan reduction product was determined on a photospectrometer (Cambridge Life Instruments) at 492 nm and standardised per 10 mm of worm.

The remaining portion of the female worm was transferred into a mortar with PBS (final volume, 2 ml), gently crushed with a pestle, and a sample transferred to a Fuchs-Rosenthal chamber (3.2 μl). A count (embryogram) was then performed by microscopy, differentiating between each intrauterine developmental stage and between normal and pathological embryos [27].

### Drugs and pharmacokinetics

UMF-078, UMF-060 and flubendazole were obtained from the MACROFIL Programme, WHO. Ammonium acetate, high-pressure liquid chromatography (HPLC)-grade methanol, diethyl ether, dimethyl sulphoxide (DMSO) and acetic acid were obtained from Fisher Scientific (Fair Lawn, NJ, USA). A C_18 _reversed-phase stainless steel column (150 × 4.6 minimum internal diameter; 5 μm particle size; Inertsil ODS-2) was supplied by Alltech (Deerfield, IL, USA).

Plasma UMF-078 and its two metabolites, UMF-060 and flubendazole, were determined by HPLC as described by Ramanathan *et al*. [28]. The detection limits of UMF-078, UMF-060 and flubendazole in plasma were 7, 5 and 7 ng/ml, respectively. The absolute recoveries of the extraction procedure were determined by comparing the peak areas obtained from extracted plasma samples with those obtained from equivalent amounts of the 3 compounds (UMF-078, UMF-060 and flubendazole) in DMSO by direct injection. For UMF-078, the mean recoveries of various concentrations ranged from 80.5 to 84.0%; for UMF-060, 88.1 to 89.6%; and for flubendazole, 97.1 to 98.6%. Intraday precision was performed by repeating the analysis of four concentration sets for UMF-078, UMF-060 and flubendazole. The coefficients of variation were <6% for all compounds and all measured concentrations were within the range ± 20% of the actual value.

### Statistical Analyses

Data were analysed using the Stata^® ^version 9 statistical package (Stata Corp., Texas, USA). The generalised linear latent and mixed models (*gllamm*) package was used for multi-level models [29]. Continuous response variables (mff, nodule size and MTT) were modelled with a normal error structure. The fixed effects of treatment group and time and their interaction were modelled for each response with a random intercept to allow for the repeat samples from the same animal. A reduced model for each response was analysed for a single time point at 24 weeks and the effect of each treatment group estimated in comparison to the control group. Assumptions of normality were checked using the qnorm plot and tested using the Wilks-Shapiro test of normality. Where the linear assumptions were not met the non parametric Kruskal-Wallis ANOVA was used which is based on ranks, however, this analysis can not include the multi-level structure of the data.

A non-compartmental analysis in WinNonlin software (Scientific Consulting, Inc., Apex, NC, USA) was used to characterize UMF-078 plasma concentration-time profiles. The terminal half-life (t_1/2_) was estimated using the last 3 or 4 data points from each curve using nonlinear regression. The area under the plasma concentration-time curve (AUC) was calculated by the linear trapezoidal rule from T = 0 to the last measured concentration (t_last_).

## Results

### Microfilariae

The geometric mean mf densities per 100 mg of skin are presented in Table 1 where the large amount of variation between animals even before trestment can be clearly seen. In the two groups treated im, there was a decline in mf density over the 72 weeks that this was monitored. This was more rapid in the higher dose im group. For the higher ia dose of 150 mg/kg, there was an initial sharp decline in mf density; however, mf density then rebounded by 24 weeks pt to approximately 100 mf per 100 mg skin (similar to the untreated controls), and this was maintained through to 72 weeks pt (final observation). The statistical analysis based on the log+1 of the raw mff counts (Table 2) shows a highly significant effect of both treatment group and time as well as an interaction between them. Only the interactions between time and the im treatment groups were highly significant (p < 0.001). At 24 weeks post treatment the results suggest that UMF-078 given at the higher drop by the im route produces a significant decline in mff compared to the controls. This appears to be a permanent decline in mf density as there is no evidence of recovery by 72 weeks.

**Table 1:** Geometric mean (3 skin snips) *O. ochengi *mf density+1 per 100 mg skin in cattle following a single treatment of UMF-078 at 150 mg/kg or 50 mg/kg and by the intramuscular or intraabomasal routes of administration.

treatment group	ID	0 wpt	1 wpt	4 wpt	12 wpt	24 wpt	42 wpt	52 wpt	72 wpt
Control	42	125.7	83.7	61.7	104.3	88.9	24.1	5.2	124.9
	46	5.1	135.8	256.5	60.3	72.7	7.8	16.2	139.3
	61	30.7	151.4	49.3	122.2	14.6	1237.0	32.9	47.0
150 mg/kg UMF-078 im	74	2298.1	238.8	0.0	0.0	0.0	0.0	-	0.8
	88	1237.6	959.4	0.0	0.0	4.3	0.0	-	0.0
	92	3069.9	713.4	111.1	10.0	14.4	1.6	-	0.0
50 mg/kg UMF-078 im	79	392.7	415.9	329.4	4.6	15.3	0.0	1.3	0.0
	80	556.8	57.6	25.6	26.1	13.4	30.6	24.0	0.0
	90	1998.6	934.5	164.7	357.6	73.0	10.0	6.2	20.8
150 mg/kg UMF-078 ia	81	76.2	138.6	5.6	18.2	142.6	77.7	-	11.5
	85	309.9	17.8	1.2	0.0	118.5	139.4	-	359.1
	91	989.5	41.4	18.9	0.0	0.0	*	*	*
50 mg/kg UMF-078 ia	82	1010.3	46.1	26.3	93.0	2.8	67.1	-	83.9
	84	3141.6	541.5	477.7	589.3	217.1	116.8	-	317.6
	93	1163.2	165.5	99.6	5.7	140.7	305.4	-	91.5

Animals lost to follow-up are indicated in the table by (*) and sample not collected by (-).

**Table 2:** Results of multilevel model for each of the variables measured.

	**Mff^+^**	**Nodule diameter^+^**	**♂/nodule^#^**	**♀MOT^#^**	**♀OD_492_^+^**
Interaction (time*trtgrp)	<0.001	<0.001	ND	ND	0.054
**Fixed effect (time)**	<0.001	<0.001	ND	ND	0.001

**Individual treatments compared to controls at 24 weeks**					
**150 mg/kg UMF 078 im**	0.005	0.002	0.001	<0.001	<0.001
**50 mg/kg UMF 078 im**	0.481	0.679	0.006	0.103	0.008
**150 mg/kg UMF 078 ia**	0.729	0.789	0.927	0.952	0.179
**50 mg/kg UMF 078 ia**	0.155	0.347	0.189	0.482	0.858

These were run using the *gllamm *procedure in STATA9 with either a linear(+) error structure and a random intercept, non parametric Kruskal Wallis ANOVA (#).

### Nodule diameter, males per nodule, female motility scores and female viability (OD_492_/10 mm)

The nodule diameter and triple assay scores are presented in Table 3. In the 150 mg/kg im-treated group, there was a rapid decline in nodule size which was already apparent by 3 weeks pt on palpation of the nodules *in vivo*. At 24 weeks pt, many nodules could only be located using the microchip scanner and they were statistically significantly smaller for the higher-dose im group compared to the controls (Table 2). After biopsy, a small calcified mass could be found in the fascia, but by 52 weeks pt no nodules could be found. From a total of 186 nodules at the start of the study none could be palpated at 52 weeks pt, demonstrating an unequivocal shrinkage and disappearance of nodules in this high-dose treatment group. In the 50 mg/kg im group, nodule size started to decline by 24 weeks pt but this was not significant (p = 0.679). At 52 weeks pt, no nodules could be palpated in one animal, but were still palpable and within the normal range in the remaining 2/3 animals. There were statistically significant treatment group, time and treatment group time interactions effects (Table 2) and this was due to both im groups having significant time interactions. Comparisons with the controls at 24 weeks showed no statistically significant difference in nodule diameter of the low dose im group or either of the groups treated by the ia route compared to the controls.

**Table 3:** Mean nodule diameters (mm), mean number of males per nodule (♂/nodule), median female motility score (♀ MOT) and mean female viability (as OD_492 _values per 10 mm length; see text) (♀ OD_492_) for *O. ochengi *recovered from cattle before and up to 52 weeks after a single dose of UMF-078 at 2 doses (50 and 150 mg/kg) and by 2 routes of administration (im = intramuscularly; ia = intra abomasally).

**Weeks**		**0**	**3**	**12**	**24**	**52**
**control**	nodule diameter	8.7	8.0	7.5	6.3	7.1
	♂/nodule	0.7	1.7	1.5	1.3	0.5
	♀MOT	2.0	2.0	2.0	2.0	1.5
	♀OD_492_	0.22	0.21	0.32	0.15	0.10
**150 mg/kg UMF 078 im**	nodule diameter	8.5	6.5	6.0	3.3	ND
	♂/nodule	0.2	0.7	0.1	0.0	
	♀MOT	2.0	1.5	0.0	0.0	
	♀ OD_492_	0.29	0.12	0.01	0.0	
**50 mg/kg UMF 078 im**	nodule diameter	7.7	7.7	6.3	5.9	5.6
	♂/nodule	1.0	0.5	0.3	0.3	0.0
	♀MOT	2.0	2.0	0.8	2.0	0
	♀OD_492_	0.31	0.13	0.06	0.11	0.17
**150 mg/kg UMF 078 ia**	nodule diameter	7.7	7.8	7.2	6.6	ND
	♂/nodule	1.5	0.5	0.5	1.0	
	♀MOT	2.0	2.0	2.0	2.0	
	♀ OD_492_	0.31	0.13	0.16	0.18	
**50 mg/kg UMF 078 ia**	nodule diameter	7.5	8.5	7.3	7.2	ND
	♂/nodule	0.5	0.3	0.8	0.6	
	♀MOT	2.0	2.0	2.0	2.0	
	♀ OD_492_	0.14	0.15	0.14	0.14	

N.B. The numbers of ♀ per nodule are not given, as 231 of the 240 nodules examined contained a single female. In addition, the male motility and MTT reduction/formazan formation data are not presented here because of the small numbers of males recovered after treatment.

ND = not done.

The numbers of males found in each nodule fluctuated considerably between time ponts even within the control group. By 24 weeks pt there were no male worms recovered from nodules treated at 150 mg/kg im and similarly at 52 weeks pt for the 50 mg/kg im group. In the other groups, males were recovered in normal numbers throughout the experiment. The counts of males count not be fit to a linear modelin gllamm and a non parametric Kruskal Wallis ANOVA was used on the ranks and did not include an adjustment for the repeated multi-level data structure. However, based on an ANOVA there was a strongly significant difference between the number of males in the control and high dose im treatment group.

The motility of female anterior ends in the 150 mg/kg im group started to decline immediately and by 12 weeks pt their motility was zero. The group treated at 50 mg/kg im showed a decline to 0.8 by 12 weeks, but recovered to a score of 2 by 24 weeks only to decline to zero at 52 weeks pt. However, these median data do not reflect the observed disappearance of worms from one animal in this group. A non-parametric Kruskal-Wallis ANOVA was used as the residuals from the *gllamm *procedure were not normal violating the assumptions of the model. This non-parametric approach did not allow the multi-level structure of the data to be included in the estimation process. There was a significant difference in female motility scores for the high dose im treatment group but no statistically significant difference between the other three groups and the controls (Table 2).

The viability (OD_492_) of females appeared to have declined in all the groups including the controls by 24 weeks pt. In the 150 mg/kg im treatment group, viability scores declined markedly after treatment and worms were no longer viable (OD_492 _< 0.1) by 12 weeks pt. In the 50 mg/kg im group there was a marked decline in viability by 12 weeks pt, but by 52 weeks mean viability had returned to pre-treatment levels (OD_492 _= 0.17) in worms obtained from intact nodules. However, in addition to the resolution of all nodules from one animal from this group, 2 of 4 nodules from a second animal treated 50 mg/kg im did not contain viable worms. At 24 weeks pt, the two im treatment groups had statistically lower viability scores compared to the controls. There was no statistically significant effect on viability of worms recovered from animals treated ia compared to controls at 24 weeks pt.

### Embryogenesis

**Figure 1:**
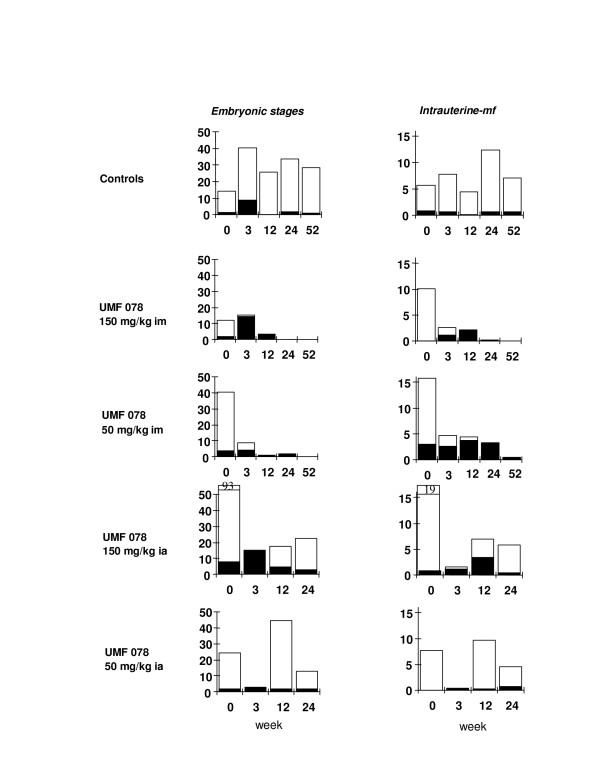
**Mean total numbers (×1000) of embryonic stages and intra-uterine mf (iu-mf) '□' overlaid with the number of abnormal/pathological stages '■' (n = 12, except at 0 weeks where n = 6).** Data represent the median percent of pathological forms for embryograms of female *O. ochengi *recovered from cattle before and up to 52 weeks after a single dose of UMF-078 at 2 dose rates (50 and 150 mg/kg) and administered by 2 routes (im = intramuscularly; ia = intraabomasally).

Embryograms are presented in Figure 1. There were both quantitative and qualitative changes in the embryograms in both im treated groups, with a greater and more rapid effect in the higher dose group. These changes were slower in onset in the fully developed intrauterine mf. In groups treated ia, there was an increase in pathological forms and decrease in total intrauterine contents at 3 weeks pt, although at later times pt, embryograms and thus reproduction returned to normal. There was a increase in the percentage of pathological embryonic stages by 12 weeks pt in both im treatment groups compared to the controls, and for intrauterine mf in the high-dose im group only compared to the controls. Comparisons between percentages of pathological stages at 24 weeks pt were not possible, as over half the nodules had no embryonic or intrauterine mf present.

### Pharmacokinetics

**Figure 2:**
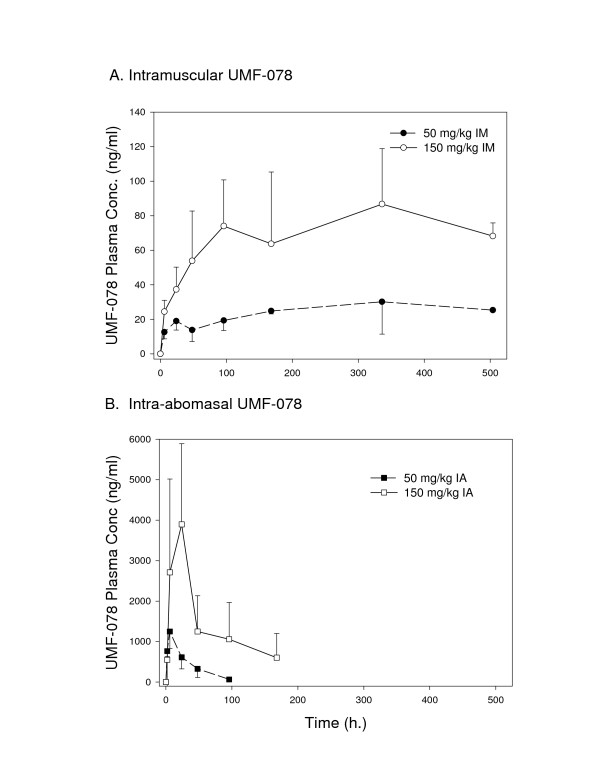
**A. Plasma concentration-time profiles for UMF-078 after intramuscular administration of 50 mg/kg, and 150 mg/kg. **B. Plasma concentration-time profiles for UMF-078 after intraabomasal administration of 50 mg/kg and 150 mg/kg). Data are means ± S.D. (n = 3).

UMF-078 absorption after im dosing was prolonged (Figure 2A), with mean T_max _of 225 and 312 hours for the 50 and 150 mg/kg dosages, respectively. The mean C_max _was 39 and 91 ng/ml for the 50 and 150 mg/kg dosages, respectively. The elimination half-life was estimated at 425 for the 50 mg/kg dose and 419 hours for the 150 mg/kg dose. The extent of absorption was highly variable, but there was a roughly proportional increase in the AUC with dose for the 50 and 150 mg/kg dosages (11,304 vs. 35,386 ng/ml × h).

UMF-078 absorption following ia dosing was variable (Figure 2B), with mean C_max _of 1638 and 3895 ng/ml for the 50 and 150 mg/kg dosages, respectively, and mean T_max _reached at about 4.7 and 24 hours, respectively. The elimination half-life was estimated at 16.9 and 38 hours for the 50 and 150 mg/kg dosages, respectively. The extent of absorption (AUC) after ia administration was highly variable, with a 6-fold increase in the AUC0-168 h when the 50 and 150 mg/kg dosages were compared (40,986 vs. 271,250 ng/ml × h).

## Discussion

The benzimidazoles are a large group of compounds used extensively in both human and veterinary medicine for the treatment of nematode infestations. UMF-078 is a modified form of flubendazole designed for parenteral injection; following which it is quickly metabolised to UMF-060 and the active parent molecule. In contrast, the standard preparation of flubendazole is insoluble and poorly absorbed across the intestinal mucosa [30]. As an aqueous suspension, it was not macrofilaricidal against *Brugia pahangi *per os [17], but when micronised the oral preparation was both macro- and microfilaricidal [18]. It has also been used successfully against *Breinlia booliati *in rats by repeated subcutaneous injection [16], although this route of administration caused abcessation and local inflammation. No such reactions were observed in the current study with UMF-078.

In this experiment, we have shown that UMF-078 (when delivered systemically) is a potent macrofilaricide against the naturally-occurring, skin-dwelling parasite, *O. ochengi *when given at 150 mg/kg im. The specific target for the drug was not investigated in this experiment, but it could act by at least two distinct (but not mutually exclusive) mechanisms. Firstly, it may cause lethal damage to the intestine by binding to the cytoplasmic microtubules of absorptive cells [31], leading to starvation and ultimately death by interference with glucose uptake. Secondly, it could induce neurotoxic effects via a predilection for neurotubules (and possibly neurofilaments), as postulated for febantel and mebendazole against the nematode *Heterakis spumosa *[32], and benomyl against the annelid *Eisenia foetida *[33]. Indeed, neurological side-effects in the mammalian host were observed after repeated dosing with UMF-078 (see below), suggesting that it may exhibit greater activity against the nervous system than other benzimidazoles. Drug effects were both dose and route dependent (with individual variation in peak levels in plasma reflected in the variation in efficacy against nodules within each group), demonstrating a requirement for sustained plasma drug concentrations, as previously reported by Court [34] and Devaney *et al*. [35].

Albendazole is currently used in combination with diethylcarbamazine or ivermectin for mass drug administration by the Global Programme to Eliminate Lymphatic Filariasis [36]. The additive or synergistic effect of this benzimidazole, if any, when used in combination with other anthelminthics for the treatment of lymphatic filariasis remains controversial, particularly with regard to macrofilaricidal efficacy [37-40]. Certainly, albendazole had no significant micro- or macrofilaricidal activity in clinical trials for human onchocerciasis conducted in the early 1990s, although it induced embryotoxic effects in female worms [41,42]. Whether the more demonstrable macrofilaricidal efficacy of UMF-078 is an intrinsic characteristic of the drug (*e.g*. due to a predilection for the nematode nervous system as discussed above) or related to enhanced systemic availability will require evaluation of albendazole against *O. ochengi*, which has not been performed to date.

The effects of UMF-078 on embryogenesis were apparent at both doses and by both routes. However, by the ia route (which was used to mimic oral administration in monograstric species such as man) the effects were transient and although embryonic and intrauterine mf were fatally damaged, there was no lasting effect on oogenesis and no accumulation of dead and dying stages in the uterus. Thus, UMF-078 disrupts embryonic development but in contrast with the avermectins [43], uterine activity is not disturbed and dead and dying mf are eliminated from the uterus and from within the nodule allowing insemination and a new reproductive cycle [44]. In our experiment, normal numbers and morphological integrity had returned by 12 and 24 weeks for the embryonic and intrauterine mf, respectively, following a single ia treatment.

In this experiment, single ia or im administration of UMF-078 did not produce any apparent acute or chronic toxic signs. However, repeated oral dosing of an aqueous suspension in dogs (WHO internal report) led to lethal neurotoxic side effects. We also observed severe neurotoxicity in cattle after two doses of UMF-078 at 150 mg/kg per os (Trees et al., 1998; and A.J. Trees, unpublished observations), and this trial had to be abandoned for welfare reasons. Data indicating that certain benzimidazoles can also induce genotoxicity ([45] and WHO internal report) and cytotoxicity [46] are of related concern, and as a result further development of UMF-078 as a macrofilaricide has been halted.

The current distribution of ivermectin has been very successful in reducing the morbidity due to *O. volvulus *[47]; however, interruption of transmission in Africa by ivermectin alone is not achievable [13,14]. This emphasises the need to upgrade the search for a practicable macrofilaricidal regimen for chemotherapy of *O. volvulus*. The best tolerated candidate drugs presently available are anti-rickettsial compounds that target endosymbiotic bacteria (*Wolbachia*) within filarial tissues [48], but these therapies have to be prolonged to achieve macrofilaricidal effects [49] and so are unsuitable for mass administration [50]. The results presented here strongly validate the potential of β-tubulin, the binding site of benzimidazoles [51], as a key molecular target for rational drug design of macrofilaricides. However, they also draw attention to potential effects on the nervous system that could extend beyond the parasite to the host., Nevertheless, benzimidazoles with an excellent safety profile (such as albendazole) should be evaluated in combination with antibiotics to determine if synergistic effects against adult worms can be achieved.

## Competing interests

The authors declare that they have no competing interests.

## Authors' contributions

BB carried out the field work, analysed the data and wrote the paper

BM analysed the data and wrote the paper

AR Conceived the study and wrote the paper

VT Conceived the study and wrote the paper

LF Carried out the pharmacokinetic analysis and wrote the paper

DE Carried out the laboratory analysis and contributed to writing the paper

AT Conceived the study, design and wrote the paper
